# Visualizing the distribution of durvalumab targeting PD-L1 using quantitative fluorescence imaging in patients with esophageal cancer

**DOI:** 10.7150/thno.136523

**Published:** 2026-09-02

**Authors:** Anne M. van der Waaij, Ymke J. van Ginkel, Pia Volkmer, Gursah Kats-Ugurlu, Henrik K. Huizinga, Sara Hone Lopez, Jacco J. de Haan, Rina Bijlsma, Laurens A. van der Waaij, Christina T. Muijs, Anne G.H. Niezink, Boudewijn van Etten, Jessie Westerhof, Massimiliano di Pietro, Dominic J. Robinson, Marjolijn N. Lub-de Hooge, Wouter B. Nagengast

**Affiliations:** 1 Department of Gastroenterology and Hepatology, University of Groningen, University Medical Centre Groningen, Hanzeplein 1, 9713 GZ Groningen, The Netherlands.; 2 Department of Pathology and Medical Biology, University of Groningen, University Medical Centre Groningen, Hanzeplein 1, 9713 GZ Groningen, The Netherlands.; 3 Department of Clinical Pharmacy and Pharmacology, University of Groningen, University Medical Centre Groningen, Hanzeplein 1, 9713 GZ Groningen, The Netherlands.; 4 Department of Medical Oncology, University of Groningen, University Medical Centre Groningen, Hanzeplein 1, 9713 GZ Groningen, The Netherlands.; 5 Department of Gastroenterology and Hepatology, Martini Hospital, Groningen, Van Swietenplein 1, 9728 NT Groningen, The Netherlands.; 6 Department of Radiation Oncology, University of Groningen, University Medical Centre Groningen, Hanzeplein 1, 9713 GZ Groningen, The Netherlands.; 7 Department of Surgery, University of Groningen, University Medical Centre Groningen, Hanzeplein 1, 9713 GZ Groningen, The Netherlands.; 8 Early Cancer Institute, Department of Oncology, University of Cambridge, Adrian Way, Cambridge CB2 0XZ, United Kingdom.; 9 Department of Otorhinolaryngology, Head and Neck Surgery, Erasmus MC Cancer Institute, Dr. Molewaterplein 40, 3015 GD Rotterdam, The Netherlands.

**Keywords:** esophageal cancer, immune checkpoint inhibitor, fluorescence imaging, durvalumab-680LT, drug distribution

## Abstract

**Rationale:**

Immune checkpoint inhibition (ICI) targeting programmed death-1 or its ligand (PD-1/PD-L1) is being explored as an addition to standard treatment for esophageal cancer (EC) to improve response rates, yet robust methods for patient selection are lacking. The predictive value of PD-L1 expression in biopsies is inconsistent due to intra- and intertumoral heterogeneity, and the impact of neoadjuvant chemoradiotherapy (CRT) on ICI response is unclear. Improving patient selection for receiving ICI therapies in patients with EC requires a better understanding of the drug’s distribution and target binding. Here, we investigated the distribution of fluorescently labelled durvalumab (durvalumab-680LT) using ultrasound-guided quantitative fluorescence molecular endoscopy (US-qFME) before and after CRT in 20 patients with EC.

**Methods:**

US-qFME included *in vivo* mucosal fluorescence imaging, spectroscopic quantification, and biopsy sampling of the tumor and healthy tissue. For dose optimization, 0, 4.5, 15, and 25 mg durvalumab-680LT were used pre-CRT.

**Results:**

Quantifying *in vivo* fluorescence revealed a dose-dependent increase in the durvalumab-680LT signal in tumor tissues pre-CRT, with the highest fluorescence intensity and highest interpatient variability in the 25-mg group (ranges: 4.5 mg: 0.0101–0.0202 mm⁻¹, 15 mg: 0.01295–0.03225 mm⁻¹, 25 mg: 0.01720–0.0514 mm⁻¹). Sixty percent of patients (n = 3 in both dose cohorts) in the 15 mg and 25 mg cohorts exhibited high durvalumab-680LT signals with a tumor-to-healthy tissue ratio ≥2. Lower fluorescence signals were measured post-CRT compared to pre-CRT. Finally, durvalumab-680LT binding to tumor cells was confirmed using fluorescence microscopy.

**Conclusions:**

US-qFME combined with durvalumab-680LT enables the visualization of durvalumab uptake in EC and reveals interpatient variability, supporting its potential future role in selecting patients for ICI therapy.

## Introduction

Esophageal cancer (EC) is one of the leading causes of cancer-related deaths worldwide 1. EC comprised two main histological subtypes: esophageal adenocarcinoma (EAC) and esophageal squamous cell carcinoma (ESCC), of which EAC is the predominant subtype in western countries 2. In the Netherlands, several valid treatment modalities are available for both types of locally advanced EC. The most common curative approach includes neoadjuvant chemoradiotherapy (nCRT) using the CROSS regimen 3 or for EAC perioperative chemotherapy using the FLOT (fluorouracil, leucovorin, oxaliplatin, and docetaxel) regimen 4, 5, both of which are typically followed by surgical resection, an extensive, invasive procedure associated with a well-documented high complication rate and mortality rate 6, 7. Alternatively, definitive chemoradiotherapy (dCRT) may be considered, particularly in patients who are not a viable candidate for surgery and/or based on tumor characteristics and patient preferences. Despite improvements in the treatment of EC, patient survival remains low 8-11. On the other hand, achieving a pathological complete response (pCR) following CRT is associated with improved long-term outcome 12, 13; however, only 16-43% of patients achieve a pCR 11. Thus, improving upon current treatment strategies is urgently needed in order to optimize clinical outcome in EC.

In recent years, immune checkpoint inhibitors (ICIs) targeted against programmed death-ligand 1 (PD-L1) and programmed cell death-1 (PD-1) were approved for use in both types of EC 14, but are not routinely used in a curative setting 15. In the recently reported MATTERHORN trial, Janjigian *et al*. showed that adding the ICI durvalumab to the FLOT regimen for gastro-esophageal junction adenocarcinoma significantly improved event-free survival compared to patients who received FLOT alone 16. At 18 months 73.2% of patients receiving durvalumab+FLOT remained event-free versus 63.6% of patients in the FLOT-only group; at 24 months, 67.4% and 58.5% of patients, respectively, were event-free. In contrast, the authors found no difference in survival among the patients receiving durvalumab when comparing high PD-L1 expression versus low PD-L1 expression 16. Although ICIs can significantly increase survival, their use is often associated with immune-related adverse events 17; therefore, identifying patients suitable for ICI therapy is crucial in order to maximize its clinical benefits while minimizing harm.

In clinical practice, assessing PD-L1 expression using the combined positive score (CPS) in biopsy samples stained by immunohistochemistry is routinely performed in order to select patients eligible for palliative treatment with ICI in addition to chemotherapy. Approximately half of both types of EC cases have high PD-L1 expression 18-21. However, not all patients with high PD-L1 expression benefit from therapy, whereas some patients with no measurable PD-L1 expression do benefit 16, 22. Tumor heterogeneity, staining variability, sampling errors, and “poor” to “fair” inter-observer and intra-observer agreement amongst pathologists can complicate patient stratification if based solely on an immunohistochemical evaluation of PD-L1 expression 23, 24.

These limitations highlight the need for new tools that can assess the entire patient rather than relying on data obtained from a single biopsy site. Such tools will improve our understanding of the distribution of ICIs and facilitate a more accurate selection of patients for the addition of PD-1/PD-L1–targeted therapies for EC, while taking into consideration both spatial and temporal heterogeneity. Here, we used a novel imaging technique called ultrasound-guided quantitative fluorescence molecular endoscopy (US-qFME). Importantly, US-qFME combines mucosal quantification using qFME with ultrasound-guided fluorescence measurements of tissue below the mucosal surface 25. Thus, US-qFME allows for the visualization and quantification of fluorescently labelled drugs, revealing valuable information regarding the drug’s distribution and binding to its target cells. In this study, we used US-qFME to measure the uptake and distribution of an ICI using fluorescently labelled durvalumab in EC patients before and after CRT. By doing so, we aim to take initial steps towards the development of an imaging-based approach that may, in the future, help improve patient selection and optimize the timing of anti-PD-L1 therapy.

## Methods

### Clinical trial design

This prospective phase I safety, feasibility, and dose-finding study was conducted at the University Medical Centre Groningen (UMCG). Initially, 21 patients with locally advanced esophageal cancer were included in the study; however, one patient did not undergo US-qFME after receiving the tracer due to logistical challenges and was therefore excluded from the study. To meet the inclusion criteria, patients had to be ≥18 years of age and have an indication for either nCRT using the CROSS regimen or dCRT. Exclusion criteria included concurrent immunosuppressive treatment and/or active autoimmune disease, esophageal endoscopic resection prior to neoadjuvant treatment, a previous infusion reaction to monoclonal antibodies, treatment with another investigational drug within 30 days prior to administration of fluorescently labelled durvalumab, and pregnancy or breastfeeding. All participating patients provided written informed consent prior to undergoing any study-related procedures. The study was approved by the UMCG’s Institutional Review Board (METc Groningen; 2022/319) and was conducted in accordance with the Dutch Act on Medical Research involving Human Subjects (WMO) and in accordance with the principles of the Declaration of Helsinki (adapted at the 64th WMA General Assembly in Fortaleza, Brazil, 2013). The trial was registered at ClinicalTrials.gov (NCT05450484).

### GMP manufacturing of durvalumab-680LT

Fluorescently labelled durvalumab (durvalumab-680LT) was developed at the good manufacturing practice (GMP) facility in the UMCG hospital pharmacy 26. In brief, commercially available durvalumab (Imfinzi, AstraZeneca, Cambridge, UK) was conjugated to the near-infrared fluorescent dye IRDye680LT (LI-COR Biosciences, Lincoln, NE) and was purified using PD-10 buffer exchange columns. Finally, durvalumab-680LT was formulated in phosphate buffer (pH 7.0) and sterile-filtered to a concentration of 1 mg/mL.

### Patient groups

This clinical trial included two separate patient groups. The first ten patients were randomly assigned to receive either 4.5 mg durvalumab-680LT (n = 5) or, to serve as negative controls who did not receive durvalumab-680LT (n = 5) group. An interim analysis was then conducted to ensure safety and assess feasibility before increasing the dose of durvalumab-680LT. Safety was assessed by monitoring the patients’ vital signs (heart rate, blood pressure, and temperature) one h after tracer administration, noting any potential side effects, including infusion-related reactions. In addition, blood samples were analyzed before and after tracer administration to evaluate any potential adverse reactions such as a change in blood count, liver function, or kidney function. Furthermore, we reviewed the patients’ medical records for up to one week after tracer administration in order to identify any delayed adverse events. Safety was defined as the absence of tracer-related serious adverse events following infusion. Feasibility was defined as a visible, quantifiable increase in tracer signal within the tumor tissue.

After confirming the safety and feasibility of administering 4.5 mg durvalumab-680LT, an additional ten patients were assigned to receive either 15 mg (n = 5) or 25 mg (n = 5) durvalumab-680LT. Decisions regarding these doses for the dose-optimization study were based on prior fluorescence molecular imaging trials conducted by our research group 27, 28. In all patients, durvalumab-680LT was administered intravenously 2-4 days prior to endoscopy. The administered doses of durvalumab-680LT were considered to have no therapeutic effect, as even the highest dose (25 mg) is 60-fold lower than the standard therapeutic dose of 1500 mg administered intravenously every 3–4 weeks. The study-related procedures are depicted in Figure 1.

### *In vivo* fluorescence molecular imaging and quantification

All patients underwent standard diagnostic procedures prior to CRT, including tumor staging via endoscopic ultrasound, followed by study-specific US-qFME measurements. Additionally, only the patients who received durvalumab-680LT (4.5 mg, 15 mg, or 25 mg) underwent a post-CRT US-qFME procedure 2-5 weeks after completing nCRT or dCRT. The post-CRT procedure was not performed in patients who experienced severe side effects. Furthermore, some patients were initially scheduled to undergo the CROSS CRT regimen; however, based on their endoscopic ultrasound findings, their treatment plan was revised to perioperative chemotherapy using the FLOT regimen. Therefore, these patients were subsequently withdrawn from the study following the first procedure. The US-qFME procedures included the following techniques: (a) high-definition white-light endoscopy (HD-WLE); (b) *in vivo* visualization of fluorescence using a near-infrared fluorescence molecular endoscopy (FME) system built in-house; (c) *in vivo* mucosal quantification using multi-diameter single-fiber reflectance/single-fiber fluorescence (MDSFR/SFF) spectroscopy; and (d) quantification using ultrasound-guided needle biopsy/single-fiber fluorescence (USNB/SFF) spectroscopy (Figure 1). In addition, biopsies were obtained for *ex vivo* analyses. During all US-qFME procedures, we assessed both the primary tumor site (PTS, defined as either the primary tumor or its location identified post-CRT) and healthy esophageal tissue (defined as tissue located at least 5 cm from the PTS, given that tissue located within 4 cm of the PTS can be exposed to radiation) 29.

The fluorescence images obtained using the fluorescence camera were used to quantify the fluorescence signal. Both MDSFR/SFF and USNB/SFF spectroscopy were used for quantification, with the raw fluorescence signal corrected for the effects of absorption and scattering parameters 30. Signals were measured at several locations both on the mucosal surface and in deeper tissue structures (i.e. within the PTS and submucosally in healthy tissue). At each location, three measurements were taken, and the average signal was calculated. To obtain representative overall outcomes for both the PTS and the healthy tissue separately, the averages were combined for each tissue type (i.e. the average from both the mucosal and ultrasound-guided measurements), and the mean value was used for further analyses.

### Immunostaining

*Ex vivo* analyses were performed on the biopsy samples to validate the *in vivo* findings and to gain insight into the microscopic distribution of durvalumab-680LT in relation to PD-L1 expression. Biopsies were obtained from areas in close proximity to the sites where the mucosal spectroscopy measurements were performed. All biopsy samples were formalin-fixed and paraffin-embedded (FFPE), and 4-μm thick sections were cut from the FFPE blocks. The tissue sections were stained with hematoxylin and eosin (H&E) for histopathological examination. PD-L1 staining was performed using the automated BenchMark ULTRA IHC/ISH system (Roche Diagnostics, Rotkreuz, Switzerland) using the OptiView DAB IHC protocol. The IHC process included automated deparaffinization, antigen retrieval, primary antibody incubation using the PD-L1 22C3 monoclonal antibody (1:50; Agilent, Dako M3653), and detection using the OptiView DAB detection kit (Roche, cat. 6396500001) in accordance with the manufacturer’s instructions. PD-L1 staining was assessed by an expert pathologist (author GK) who was blinded with respect to the fluorescence results, providing a combined positive score (CPS). The CPS was calculated by dividing the total number of PD-L1–positive tumor cells and inflammatory cells (lymphocytes and histiocytes, but not including neutrophilic granulocytes, eosinophilic granulocytes, or plasma cells) by the total number of tumor cells, multiplied by 100 31. PD-L1 expression was classified as low, intermediate, or high for CPS scores < 1, 1-5, and > 5, respectively. In cases in which no tumor could be identified in the biopsy specimens, no CPS score was obtained.

### Fluorescence microscopy

*Ex vivo* fluorescence microscopy was performed in order to visualize the microscopic distribution of durvalumab-680LT in a section adjacent to the section used for PD-L1 IHC. In brief, the 4-μm section was stained with DAPI (1:1000) using a standard protocol, and durvalumab-680LT fluorescence was measured using an SP8 X confocal microscope with a 40× oil-immersion objective (Leica Microsystems, Wetzlar, Germany) using 670-nm light for excitation; the signal was detected at 695-800 nm.

### Stability of the durvalumab-680LT tracer

Sodium dodecyl-sulfate polyacrylamide gel electrophoresis (SDS-PAGE) was performed on fresh biopsy samples obtained from one patient in the 15-mg group in order to determine the stability of durvalumab-680LT in the tissue. In brief, post-CRT PTS and healthy biopsy tissues were lysed on ice using M-PER (Mammalian Protein Extraction Reagent; Thermo Fisher Scientific) containing 1% (v/v) Halt phosphatase inhibitor cocktail/Halt protease inhibitor single-use cocktail (Thermo Fisher Scientific) for at least 30 min in accordance with the manufacturer’s instructions. The lysates were then separated on a TGX stain-free 4-15% SDS-PAGE gel (Bio-Rad, cat. 64543962) using a running buffer consisting of Trizma base, glycine, and 20% (w/v) SDS at 60 V for 2 h. Two protein markers ranging from 10-190 kDa and 4.6-300 kDa (Fischer Scientific, cat. BMA50550) were included as size markers. In addition to the biopsy lysates, both durvalumab-680LT and unlabeled durvalumab were loaded on the gel as positive and negative controls, respectively. The gel was scanned using an Odyssey CLx flatbed scanner, and fluorescence was visualized at 700 nm. The gel was then incubated overnight in Coomassie blue to visualize the total protein content.

### *In vitro* experiment to visualize durvalumab-680LT and confirm PD-L1 antibody binding specificity

RKO cells (a human colorectal cancer cell line) were cultured in RPMI 1640 medium containing 10% (v/v) fetal calf serum and 1% penicillin/streptomycin/fungizone at 37 °C in humidified air containing 5% CO_2_. Durvalumab-680LT (50 nM) was added to the culture medium and the cells were incubated for 24 h, after which the cells were harvested, fixed, and embedded in 1.8% agar. The next day, the agar piece containing the cells was embedded in a paraffin block, and 4-μm sections were cut. To visualize the durvalumab-680LT signal, one section was deparaffinized and stained with DAPI (1:1000) using a standard protocol. Imaging was performed using an SP8 X confocal microscope with a 40× oil-immersion objective. Durvalumab-680LT was excited at 670 nm, and the signal was detected at 695-800 nm. An adjacent 4-μm section was stained for PD-L1 as described above.

### Statistical analyses

All data were treated as being non-normally distributed due to the relatively small sample size. As our primary aim was to visualize differences between individual patients, only descriptive statistics were used. Except where indicated otherwise, data are presented as individual values and/or the median value with range (minimum and maximum). The tumor-to-healthy tissue ratio (THR) was calculated by dividing the fluorescence value measure at the PTS by the fluorescence value in healthy tissue. *Ex vivo* scoring was qualitatively assessed, and immunostaining and fluorescence microscopy results were compared. Prism 9 (GraphPad Software Inc., San Diego, CA) was used to perform the statistical analyses and to create the data plots.

## Results

In total, 21 patients with locally advanced EC were initially included from April 2023 through October 2024 (Figure 2). One patient was excluded after receiving the tracer, as no procedures were performed due to logistical limitations; thus, 20 patients were included in the study. One adverse event was reported in one patient in the 25 mg group during the trial. Specifically, this patient experienced chest pain; however, because this symptom was present prior to tracer administration, this event was considered unlikely to be related to the durvalumab-680LT infusion. The patient and disease characteristics are summarized for each dose group in Table 1. Eighteen of the 20 patients were diagnosed with adenocarcinoma, and two patients were diagnosed with squamous cell carcinoma. Moreover, 18 patients had a cT3 tumor, while lymph node stage varied in all four dose groups. Fifteen patients underwent nCRT, and three patients were treated with dCRT; the remaining two patients received neoadjuvant chemotherapy. The second (i.e. post-CRT) US-qFME procedure was only performed in the 4.5-mg, 15-mg, and 25-mg groups; moreover, two patients receiving FLOT and four patients with persistent CRT-related side effects were withdrawn prior to the post-CRT procedure and therefore did not receive a second infusion of durvalumab-680LT (Figure 2).

### *In vivo* fluorescence molecular imaging and quantification

Qualitative assessment of the real-time *in vivo* fluorescence signals revealed specific uptake of durvalumab-680LT in the primary tumor site (PTS) compared to healthy tissue in both the 15 mg and 25 mg dose groups. In contrast, the fluorescence signals in the 4.5 mg group were too low to assess, and the signals in the 0 mg group were negligible in both the PTS and healthy tissues (Figure 3A).

Quantification of the *in vivo* fluorescence signals (Table 2) supports our qualitative assessment, revealing a dose-dependent increase in the durvalumab-680LT signals within the PTS. The highest median fluorescence signal was in the 25-mg group, with a median *Q*.μ_a_^f^ value of 0.0438 mm^-1^, compared to median values of 0.0200 and 0.0291 mm^-1^ in the 4.5-mg and 15-mg groups, respectively. Additionally, the highest maximum signal and the largest range were measured in the 25-mg group, with *Q*.μ_a_^f^ ranging from 0.0101 to 0.0202 mm^-1^, 0.0130 to 0.0323 mm^-1^, and 0.0172 to 0.0514 mm^-1^ in the 4.5-, 15-, and 25-mg groups, respectively. With respect to the signal measured in healthy tissues, we measured median *Q*.μ_a_^f^ values of 0.0133 mm^-1^, 0.0143 mm^-1^, and 0.0206 mm^-1^ in the 4.5-, 15-, and 25-mg groups, respectively (Table 2). Moreover, the THR (tumor-to-healthy tissue ratio) was ≥2 in three out of five patients (60%) in both the 15-mg and 25-mg groups (Figure 3C, right). Compared to pre-CRT, we measured smaller median signals and smaller *Q*.μ_a_^f^ ranges in the post-CRT analysis in all three groups, with values ranging from 0.0160 to 0.0170 mm^-1^, 0.0191 to 0.0236 mm^-1^, and 0.0230 to 0.0275 mm^-1^ in the 4.5-, 15-, and 25-mg groups, respectively (Figure 3C, left and Table 2). The signals measured the PTS tissue in individual patients decreased in patients with a relatively high pre-CRT signal (Figure 3C, right). When comparing the maximum signals measured in PTS tissue, we found a 1.5-fold decrease from pre-CRT to post-CRT in the 15-mg group and a 1.9-fold decrease in the 25-mg group. The signals in the control patients were negligible in both the PTS and healthy tissues, with median *Q*.μ_a_^f^ values of 0.0013 mm^-1^ and 0.0007 mm^-1^, respectively (Figure 3B and Table 2). Finally, all THRs measured post-CRT were < 2, with all patients except one having a lower post-CRT ratio compared to their corresponding pre-CRT ratio (Figure 3C, right).

### Stability of the durvalumab-680LT tracer

The stability of durvalumab-680LT in mucosal biopsies was demonstrated using SDS-PAGE, showing that durvalumab-680LT in post-CRT biopsy samples was the same size as pure durvalumab-680LT loaded as a positive control; no bands were visible for unlabeled durvalumab (Figure S1). Furthermore, consistent with our *in vivo* data, the durvalumab-680LT band was stronger in the PTS sample than in the healthy tissue sample (Figure S1).

### Immunostaining

Next, we examined whether the presence of durvalumab-680LT in the biopsy samples interfered with standard PD-L1 immunohistochemical staining. We found that RKO cells incubated with durvalumab-680LT had robust PD-L1 antibody binding (Figure S2).

We then used the biopsy samples to calculate a CPS for each patient based on PD-L1 expression. In the 4.5-mg group, two patients had a CPS < 1, two patients had a CPS ranging from 1–5, and one patient’s biopsy was deemed non-assessable for PD-L1 expression, as no tumor cells were found in the biopsy. In the 15-mg group, three patients had a CPS < 1, one patient had a CPS > 5, and no CPS score was obtained for one patient’s biopsy. Finally, in the 25-mg group, two patients had a CPS < 1, two patients had a CPS ranging from 1–5, and one patient had a CPS > 5.

### Correlation between the *in vivo* results and *ex vivo* results

We then compared the *in vivo* spectroscopy fluorescence values measured at the pre-CRT PTS with *ex vivo* PD-L1 expression measured using the CPS scoring system, and found that the patients in each group with the lowest median spectroscopy value all had a CPS score < 1 (Table 3). In addition, in each dose group one patient had a relatively high median fluorescence value though a CPS score < 1. Finally, all patients with an intermediate (1–5) or high (> 5) CPS score had relatively high median fluorescence values (Table 3).

### Fluorescence microscopy

Lastly, we performed confocal fluorescence microscopy in the pre-CRT PTS biopsy samples in order to visualize the distribution of durvalumab-680LT within the tissues. We observed high uptake of durvalumab-680LT in patients in both the 15-mg and 25-mg groups, but very low fluorescence in patients in the 4.5-mg and control groups (Figure 4A). Consistent with the pattern observed *in vitro* (Figure S2), we found cell membrane staining of durvalumab-680LT in the biopsy samples (Figure 4B), suggesting PD-L1 binding at the cell membrane. Notably, we observed clear durvalumab-680LT fluorescence in the pre-CRT PTS biopsy samples obtained from all ten patients in the 15-mg and 25-mg groups.

Comparing the durvalumab-680LT fluorescence signal with the PD-L1 staining pattern revealed strong tracer fluorescence signal in regions with tumor cells expressing high levels of PD-L1 (Figure 4B, top row). Notably, the durvalumab-680LT signal was also detected in cancer cells with little or no PD-L1 expression (Figure 4B, bottom row).

## Discussion

This study is the first to visualize and quantify the distribution of intravenously administered fluorescently labelled durvalumab – an ICI that targets PD-L1 – both *in vivo* and *ex vivo* in patients with locally advanced EC. Notably, we also provide the first data regarding CRT-induced changes in durvalumab-680LT distribution by comparing the fluorescence signals measured before and after neoadjuvant chemoradiotherapy in patients with EC. Moreover, we show that US-qFME can be used to visualize and quantify durvalumab-680LT uptake in both the esophageal PTS and in healthy tissue, showing clear variability among patients. Importantly, this approach offers a more objective alternative to conventional PD-L1 CPS scoring, thereby potentially helping guide the selection of patients for ICI therapy in the future.

The addition of durvalumab to neoadjuvant therapy is promising and relevant, as demonstrated by the recent phase III MATTERHORN trial, which showed that perioperative durvalumab combined with FLOT chemotherapy significantly improved both event-free survival and overall survival in patients with resectable gastric and gastroesophageal junction adenocarcinoma, irrespective of PD-L1 expression. However, these findings do not indicate that all patients benefit equally from ICI treatment as more than one-third of patients had disease progression, recurrence or death 16. Thus, a substantial proportion of patients is still unlikely to respond, while being exposed to potential toxicity and unnecessary healthcare costs. Therefore, patient selection for ICI therapy remains an important goal and confirming that the drug reaches its intended target is needed in order to improve patient selection. Currently, PD-L1 expression measured in biopsy samples is widely used to select patients for ICI-based therapy; however, because tumor heterogeneity is not necessarily reflected accurately in biopsies due to sampling limitations, this method has not led to reliable patient selection 18, 19, 23.

Previous PET imaging studies showed that high uptake of PD-L1–targeted tracers can predict the response to PD-L1 inhibitors in both gastroesophageal adenocarcinoma and several other cancer types 32, 33. These findings support the use of molecular imaging – which provides information regarding the entire tumor – as a viable alternative to biopsy-based tests for identifying patients who are likely to benefit from PD-L1–targeted treatment. However, PET imaging has several disadvantages such as the need for post-processing, radiation burden, relatively low resolution, and the inability to assess intra-tumoral heterogeneity or obtain biopsies during imaging. In contrast, US-qFME provides real-time visualization of the fluorescent tracer both during *in vivo* endoscopy and in *ex vivo* microscopy analyses. However, endoscopic ultrasound is an invasive – albeit minimally invasive – procedure, whereas PET imaging is completely non-invasive. Here, we combined US-qFME with durvalumab-680LT in order visualize and quantify the tracer’s signal in the esophageal PTS, revealing a dose-dependent increase in fluorescence. The 25-mg group showed a broader range of tumor tissue signals than the other dose groups, reflecting greater interpatient variability in drug uptake. Although the higher doses also increased signal intensity in healthy tissue, leading to similar tumor-to-healthy ratios in the 15-mg and 25-mg groups, the wider signal distribution in the 25-mg group improved our ability to differentiate drug uptake between patients to get better insight in drug distribution and possibly tumor biology. In addition, *ex vivo* analyses using fluorescence microscopy showed clear durvalumab-680LT signals in the 25-mg group, whereas signals where less distinct at the lower dosages. We therefore selected 25 mg for further analysis and found relatively high uptake of durvalumab-680LT in three out of five patients.

Interestingly, the *in vivo* fluorescence signals were high in all patients with high PD-L1 expression, but was also high in one patient per group with low expression of PD-L1. Similar discrepancies were reported in previous studies on PD-L1–targeted PET imaging, in which intratumoral tracer distribution only partially aligned with the PD-L1 staining patterns measured using IHC 34. These findings suggest that *in vivo* fluorescence imaging and quantification of durvalumab-680LT may identify a larger subset of patients as being eligible for ICI therapy, as they directly confirm durvalumab binding to tumor cells rather than relying solely on PD-L1 measurements using IHC. An important explanation for the discrepancy between *in vivo* signals and *ex vivo* CPS scores is the difference in sampling. PD-L1 expression is known to be spatially heterogeneous within tumors 23, 24, and CPS scoring is typically based on limited biopsy material, which may not adequately represent the entire tumor. In contrast, *in vivo* fluorescence imaging assesses tracer distribution across a larger tumor area, potentially providing a more comprehensive representation of overall target availability. Furthermore, an explanation for the discrepancy between US-qFME and IHC outcomes may be differences in IHC-based immune phenotypes. Different IHC assays can produce conflicting results; for example, comparison of the SP142 and SP263 clones showed discordant findings in over 40% of the samples 32.

To gain more detailed information regarding the distribution of durvalumab-680LT, we performed fluorescence microscopy and compared the fluorescence signals to PD-L1 staining. Consistent with previous studies highlighting the challenges of assessing PD-L1 expression due to its spatial heterogeneity 23, we observed heterogenous PD-L1 expression within our biopsy samples using IHC. In addition, fluorescence microscopy revealed that the durvalumab-680LT signal is localized to the surface of tumor cells in both PD-L1–high and PD-L1–low regions; this finding suggests that both *in vivo* and *ex vivo* imaging of durvalumab-680LT reflects the tissue distribution and tumor-binding properties of durvalumab more accurately than measuring PD-L1 expression* ex vivo* using IHC. In addition, both abundant durvalumab-680LT fluorescence and clear PD-L1–positive IHC staining were observed in cell types not included when calculating the CPS score, for example eosinophils, neutrophils and plasma cells. Nevertheless, previous studies linked PD-L1-positive neutrophils with the patient’s response to ICI 35, 36, suggesting that measuring PD-L1 expression in neutrophils – and possibly other immune cell types – may provide added value in predicting the response to ICI therapy.

In addition to suboptimal patient selection, the optimal timing of ICI treatment in relation to CRT remains unknown. Current clinical trials are investigating the concurrent administration of ICI with chemotherapy and/or CRT. Moreover, previous studies found that priming the tumor with CRT may lead to more abundant infiltration of immune cells and higher expression of PD-L1 in the tumor microenvironment 37-39, although another study found decreased tumor expression of PD-L1 after CRT 40. In our study, we observed a decrease in durvalumab-680LT fluorescence in post-CRT patients who had a relatively high durvalumab-680LT signal in the PTS prior to CRT. One hypothesis to explain this finding is that the reduction in fluorescence observed in these patients with high pre-CRT levels may reflect tumor eradication. In addition, CRT-induced changes in the tumor microenvironment may also affect tracer uptake independently of tumor burden. Furthermore, CRT can induce structural and functional changes in tumor vasculature, including alterations in perfusion and permeability, thereby affecting the delivery and distribution of systemically administered tracers 41. These findings suggest that the optimal timing for ICI therapy may actually be prior to performing CRT, providing a better outcome compared to starting ICI therapy after CRT.

Our study has several limitations that warrant discussion. First, since immunotherapy is currently not recommended as a treatment option for patients with locally advanced esophageal cancer in the Dutch guidelines, none of our patients received ICI treatment; we are therefore unable to examine the correlation between durvalumab-680LT uptake and treatment response. We therefore propose conducting a larger study using this technique in order to assess patient suitability for ICI, in which patients will receive ICI therapy after undergoing US-qFME. Furthermore, during the course of our study, the results of the ESOPEC trial (ClinicalTrials.gov: NCT02509286) were published, leading to an update of the Dutch treatment guidelines 42. As a result, perioperative FLOT chemotherapy has partly become the new standard of care for patients with locally advanced esophageal cancer and suspected lymph node metastasis. In our study, patients who received FLOT therapy were excluded from the second US-qFME procedure, as CROSS CRT was the prevailing standard at the time. However, given the recent changes in clinical practice, future studies should also focus on evaluating imaging outcomes in patients treated with perioperative FLOT, particularly given that chemotherapy affects the immune cell composition differently than CRT. Finally, due to the low number of patients we were unable to investigate potential differences in fluorescence signals between patient subgroups, such as between ESCC and EAC patients.

Our approach for using US-qFME to distinguish between patients with high versus low drug uptake has potential applications beyond the current study. Here, we used a PD-L1–targeted tracer conjugated to a fluorescent dye emitting at ~700 nm. During the course of this study, our group developed an additional ICI tracer, nivolumab-800CW, which targets PD-1 and emits light at ~800 nm. These two tracers are currently being combined in an ongoing clinical multispectral imaging trial in esophageal carcinoma (ClinicalTrials.gov: NCT07196384), using dual-wavelength imaging to visualize both PD-L1 and PD-1. This strategy will allow us to visualize and quantify PD-L1 expression in tumor cells while simultaneously measuring PD-1 expression in infiltrating immune cells, providing a comprehensive assessment of the tumor-immune landscape and get insight in spatial and temporal changes. These novel insights can shed new light on the mechanism of action of ICIs and enable the selection of patients suitable for ICI therapy. In addition, a similar approach can be used to study other targeted therapies such as treatments targeted against HER2 (human epidermal growth factor receptor 2) and PARP (poly (ADP-ribose) polymerase) proteins. Finally, fluorescence molecular imaging may have applications beyond endoscopy, including its use in ultrasound-guided interventions and other types of scopic procedures in various fields such as in pulmonology and urology.

In conclusion, our results demonstrate the feasibility of our novel approach combining US-qFME with fluorescently labelled durvalumab to gain insights into the interpatient variability of durvalumab-680LT uptake in both the PTS and healthy tissue in patients with EC. Furthermore, we show that the durvalumab-680LT signal is reduced after CRT. This exploratory work provides a first step towards developing an imaging strategy that, in the future, may support treatment stratification and timing of ICI therapy.

## Supplementary Material

Supplementary figures.

## Figures and Tables

**Figure 1:**
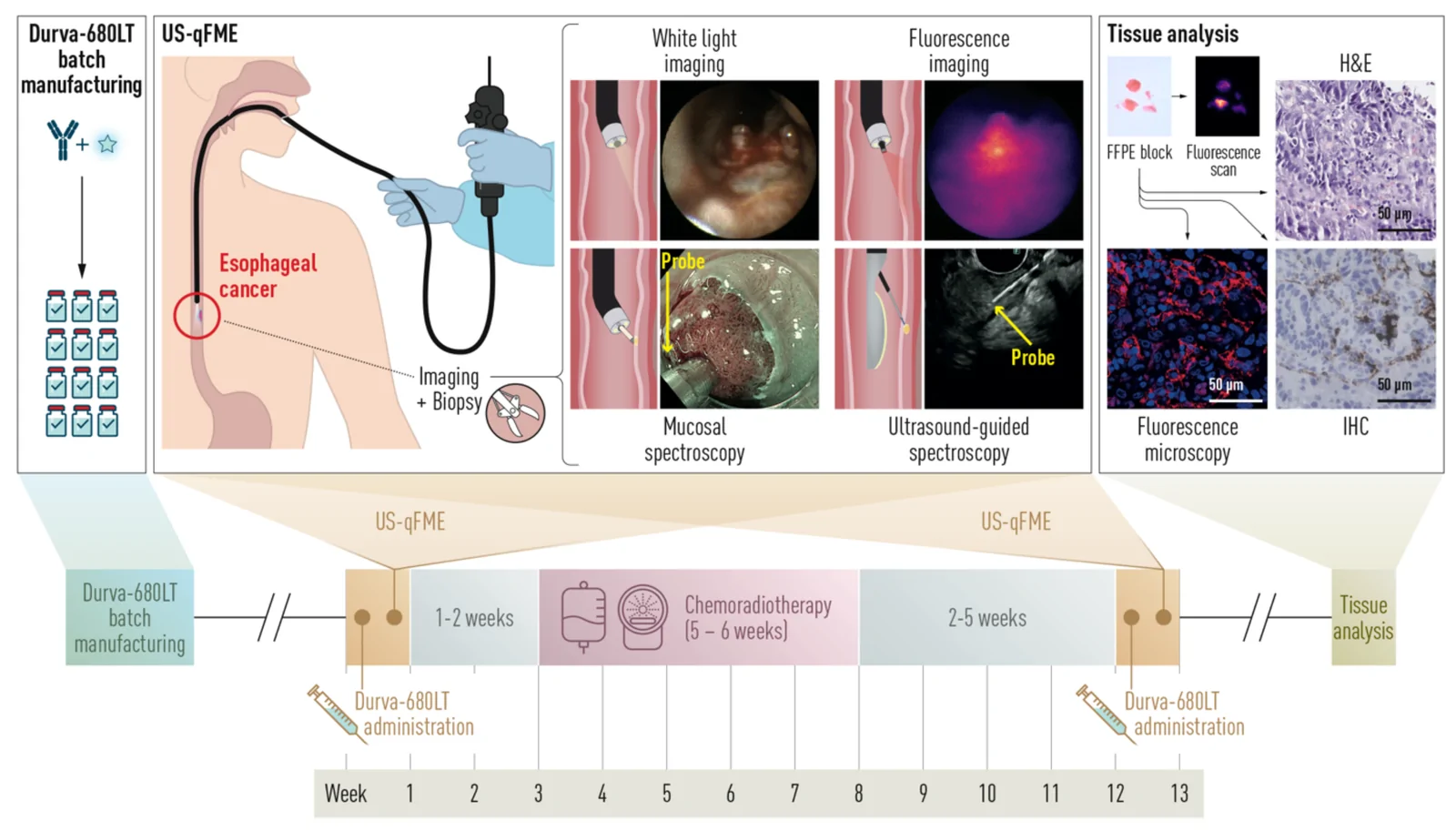
Overview of the procedures performed in this study. Fluorescently labelled durvalumab (durvalumab-680LT) was manufactured in-house using GMP standards and administered intravenously 2-4 days prior to the US-qFME procedure. US-qFME, including white-light imaging, qualitative assessment using fluorescence imaging, and quantification using mucosal and ultrasound-guided spectroscopy measurements, was used to visualize and quantify the distribution of durvalumab-680LT, and tissue biopsies were obtained for tissue analysis. Patients then underwent CRT for 5-6 weeks in accordance with standard clinical guidelines; 2-5 weeks after the completion of CRT, the patients returned for a second infusion of durvalumab-680LT and a second US-qFME procedure. Durvalumab-680LT fluorescence was visualized and quantified *ex vivo* using FFPE blocks prepared from the biopsies obtained during US-qFME. Finally, 4-μm tissue sections were used to assess the presence of tumor cells based on H&E staining, to measure PD-L1 expression using IHC, and to visualize microscopic durvalumab-680LT distribution using fluorescence microscopy. Durva-680TL: durvalumab-680LT; FFPE: formalin-fixed, paraffin-embedded; GMP: good manufacturing practice; H&E: hematoxylin and eosin; IHC: immunohistochemistry; PD-L1: programmed death-ligand 1; US-qFME: ultrasound-guided quantitative fluorescence molecular endoscopy.

**Figure 2:**
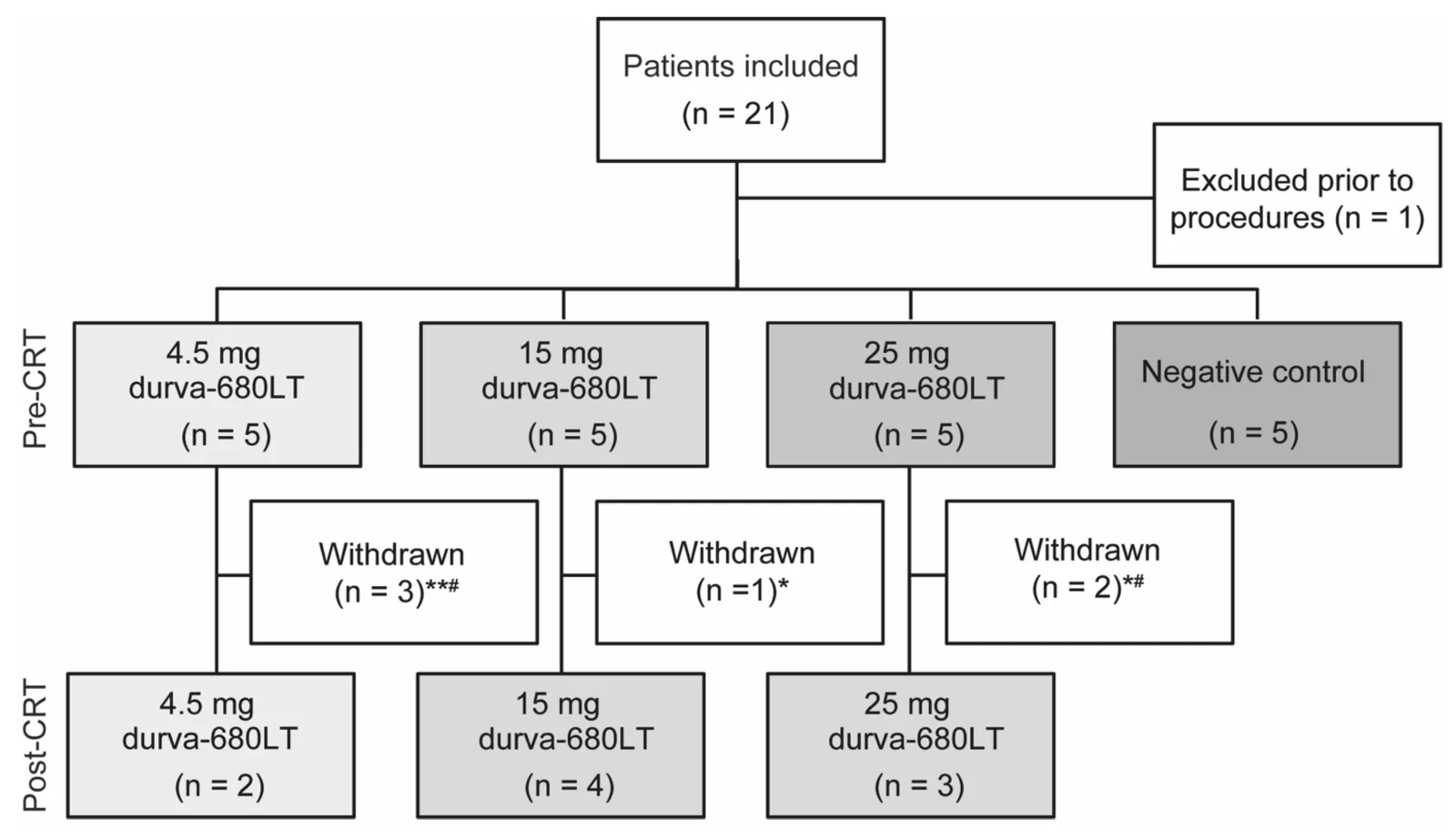
Schematic diagram depicting the various patient groups. In total, 21 patients were initially included in the study; however, one patient was excluded prior to the procedures due to logistical constraints. Thus, 15 patients were assigned to receive an infusion containing 4.5, 15, or 25 mg durvalumab-680LT. Negative control patients did not receive an infusion (n = 5 patients per group). Six patients were withdrawn prior to the second infusion because they either received FLOT (indicated by ^#^) or had persistent side effects after receiving CRT (indicated by *). CRT: chemoradiotherapy; durva-680LT: durvalumab-680LT.

**Figure 3:**
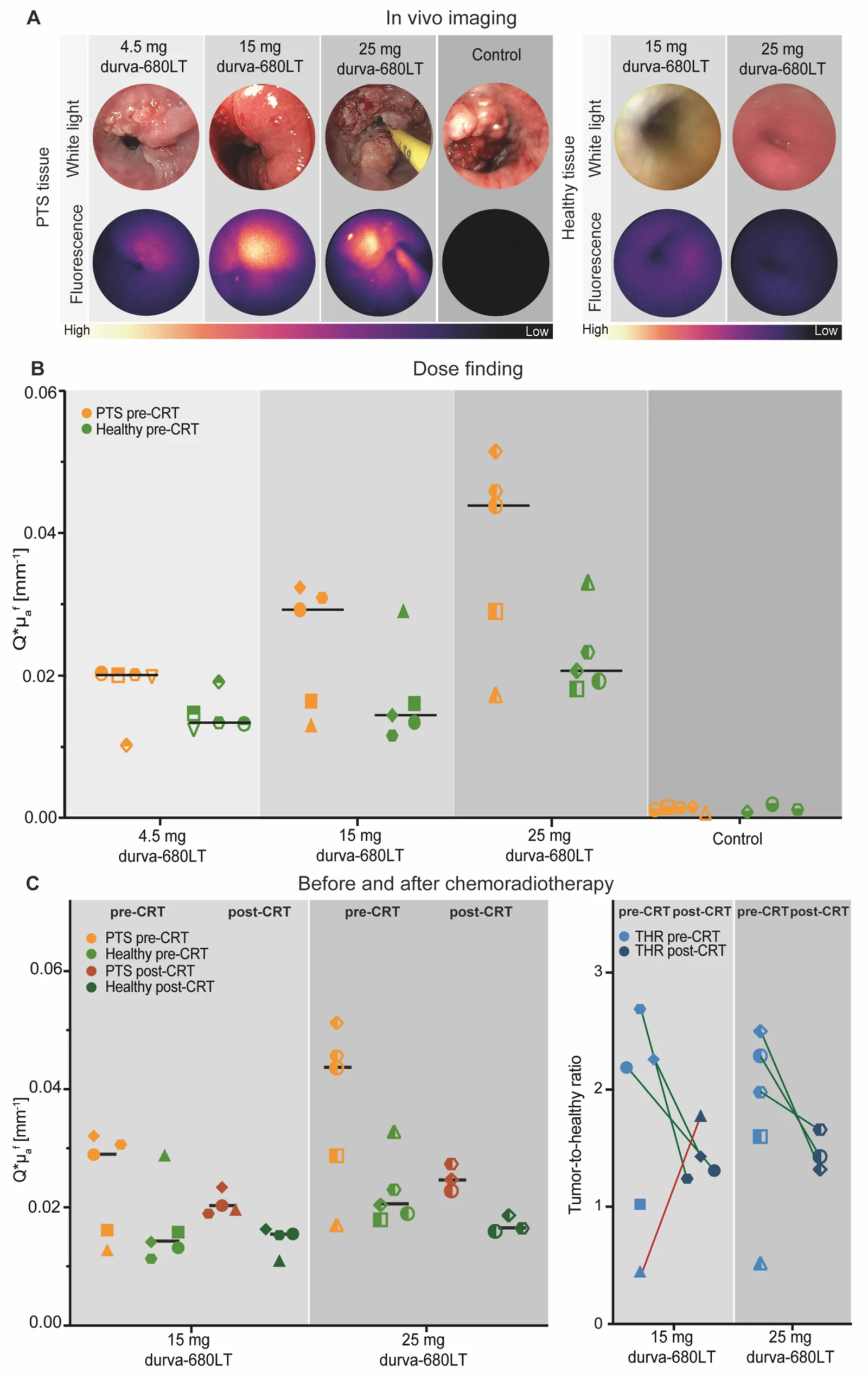
*In vivo* visualization and quantification of durvalumab-680LT fluorescence measured in both tumor tissue (PTS) and healthy esophageal tissue. (A) Representative white-light endoscopy images (top row) of PTS (left) and healthy (right) tissues are shown along with their corresponding fluorescence images (bottom row) for the indicated dose groups; the control patients received no durvalumab-680LT. Note that the fluorescence images are scaled in relation to one another to allow direct qualitative inter-image comparisons. (B) Median spectroscopy-derived fluorescence values in each group measured in PTS and healthy tissues prior to chemoradiotherapy (pre-CRT). Each symbol represents an individual patient. (C) Median fluorescence values measured in PTS and healthy tissues in the 15- and 25-mg groups before and after CRT; for comparison purposes only, the pre-CRT data are reproduced from panel B. Each symbol represents an individual patient. Shown on the right is the tumor-to-healthy tissue ratio measured in the indicated groups before and after CRT. CRT: chemoradiotherapy; durva-680LT: durvalumab-680LT; PTS: primary tumor site; THR: tumour-to-healthy tissue ratio.

**Figure 4:**
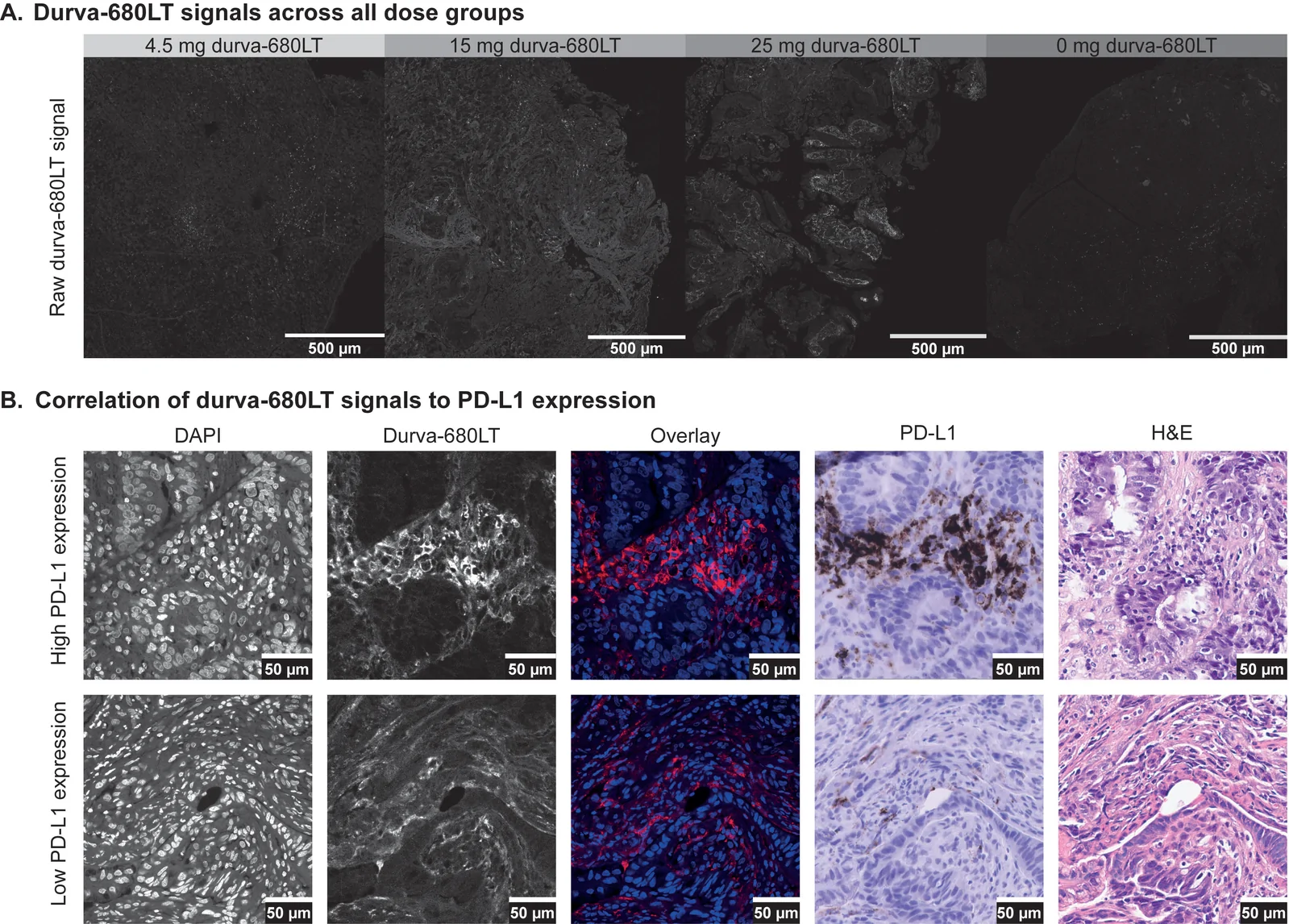
*Ex vivo* imaging of biopsy samples obtained from pre-RCT patients. (A) Representative images of raw durvalumab-680LT fluorescence in patients in the 4.5-, 15-, 25-, and 0-mg groups. (B) Magnified images of a biopsy sample obtained from a patient in the 25-mg group, showing (from left to right): nuclei stained with DAPI; the raw durvalumab-680LT fluorescence signal; a colored overlay of the DAPI (blue) and durvalumab-680LT (red) channels; PD-L1 IHC staining on an adjacent slide; and an H&E-stained adjacent slide. The images in the top row show the durvalumab-680LT signal in tumor cells that are PD-L1–positive based on IHC. The images in the bottom row show that the durvalumab-680LT signal is visible in tumor cells, with no PD-L1 staining visible in the same region. Durva-680LT: durvalumab-680LT; H&E: hematoxylin and eosin; IHC: immunohistochemistry; PD-L1: programmed death-ligand 1.

**Table 1:** Patient and disease characteristics per durvalumab-680LT dose group

Characteristic	4.5 mg *n = 5*	15 mg *n = 5*	25 mg *n = 5*	0 mg *n = 5*
Median age, years [range]	62 [41-75]	65 [46-72]	70 [62-74]	66 [49-80]
Male, n (%)	3 (60%)	5 (100%)	5 (100%)	5 (100%)
Pathology				
Adenocarcinoma, n (%)	4 (80%)	5 (100%)	5 (100%)	4 (80%)
Squamous cell carcinoma, n (%)	1 (20%)	0 (0%)	0 (0%)	1 (20%)
Staging^†^				
*cT-stage*				
T1, n (%)	0 (0%)	0 (0%)	0 (0%)	0 (0%)
T2, n (%)	1 (20%)	1 (20%)	0 (0%)	0 (0%)
T3, n (%)	4 (80%)	4 (80%)	5 (100%)	5 (100%)
T4, n (%)	0 (0%)	0 (0%)	0 (0%)	0 (0%)
*cN-stage*				
N0, n (%)	2 (40%)	4 (80%)	4 (80%)	0 (0%)
N1, n (%)	1 (20%)	1 (20%)	1 (20%)	2 (40%)
N2, n (%)	2 (40%)	0 (0%)	0 (0%)	2 (40%)
N3, n (%)	0 (0%)	0 (0%)	0 (0%)	1 (20%)
Treatment				
*Regimen*				
nCRT, n (%)	4 (80%)	5 (100%)	4 (80%)	2 (40%)
dCRT, n (%)	1 (20%)	0 (0%)	0 (0%)	2 (40%)
nCT, n (%)	0 (0%)	0 (0%)	1 (20%)	1 (20%)
*Radiation*				
Protons, n (%)	4 (80%)	4 (80%)	4 (80%)	2 (40%)
Photons, n (%)	0 (0%)	1 (20%)	0 (0%)	2 (40%)
Photons/protons combined, n (%)	1 (20%)	0 (0%)	0 (0%)	0 (0%)
No radiation	0 (0%)	0 (0%)	1 (20%)	1 (20%)
Clinical response after treatment^‡^				
Partial response, n (%)	3 (60%)	4 (80%)	3 (60%)	4 (80%)
Complete response, n (%)	2 (40%)	1 (20%)	2 (40%)	1 (20%)

dCRT: definitive chemoradiotherapy; nCRT: neoadjuvant chemoradiotherapy; nCT: neoadjuvant chemotherapy. ^†^Pre-treatment staging was determined at a multidisciplinary meeting and was based on both endoscopy with biopsies and PET-CT. ^‡^Response was assessed at a multidisciplinary meeting prior to surgery based on PET-CT and (when indicated) endoscopy with biopsies.

**Table 2:** Summary of the *in vivo* durvalumab-680LT fluorescence signals measured in each group

Dose (mg)	Median signal *Q*.μ_a_^f^ (mm^-1^)	Minimum signal *Q*.μ_a_^f^ (mm^-1^)	Maximum signal *Q*.μ_a_^f^ (mm^-1^)
PTS (Pre-CRT)			
0	0.0013	0.0006	0.0014
4.5	0.0200	0.0101	0.0202
15	0.0291	0.0130	0.0323
25	0.0438	0.0172	0.0514
PTS (Post-CRT)			
0^*^	NA	NA	NA
4.5	0.0167	0.0160	0.0170
15	0.0201	0.0191	0.0236
25	0.0249	0.0230	0.0275
Healthy tissue (Pre-CRT)			
0^*^	0.0007	0.0000	0.0018
4.5	0.0133	0.0123	0.0190
15	0.0143	0.0115	0.0290
25	0.0206	0.0181	0.0330
Healthy tissue (Post-CRT)			
0^*^	NA	NA	NA
4.5	0.0121	0.0118	0.0124
15	0.0156	0.0112	0.0165
25	0.0166	0.0161	0.0188

CRT: chemoradiotherapy; NA, not applicable; PTS: primary tumour site ^*^Fluorescence was measured in the 0-mg group pre-CRT, but not post-CRT.

**Table 3:**
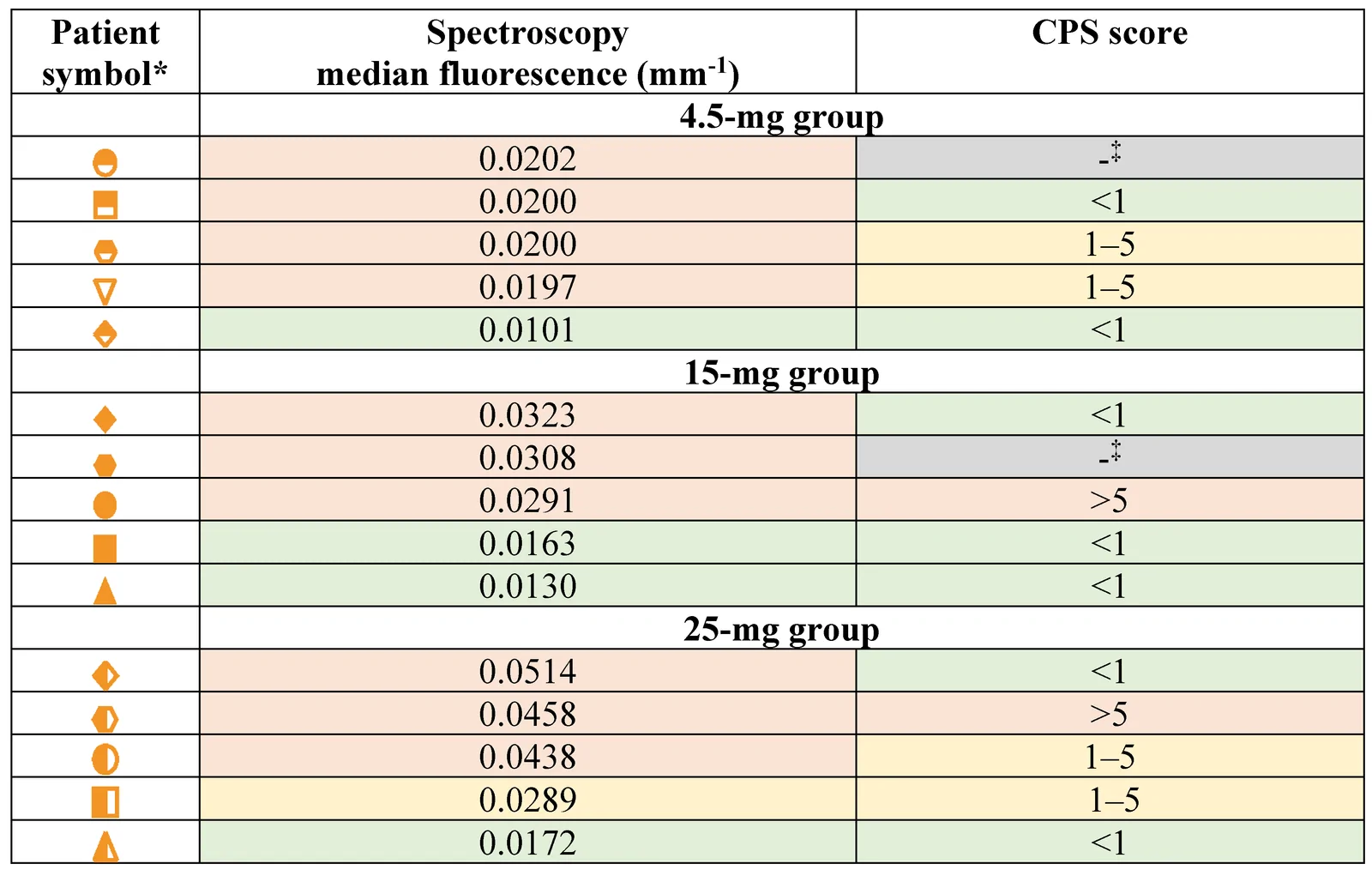
Summary of *in vivo* durvalumab-680LT fluorescence measured using spectroscopy and *ex vivo* CPS scores measured pre-CRT at the PTS in all 15 patients.

CPS: combined positive score; PTS: primary tumour site Cells shaded in green, yellow, and red indicate low, intermediate, and high values respectively. * The symbols correspond to the patients shown in Fig. 3. ^‡^ These patients’ CPS could not be determined, as no tumour cells were found in their biopsy samples.

## Data Availability

Data will be provided upon reasonable request.

## Abbreviations

CPS: combined positive score
CRT: chemoradiotherapy
dCRT: definitive chemoradiotherapy
durva-680LT: durvalumab-680LT
EC: esophageal cancer
FFPE: formalin-fixed, paraffin-embedded
FME: fluorescence molecular endoscopy
GMP: good manufacturing practice
HD-WLE: high-definition white-light endoscopy
HER2: human epidermal growth factor receptor 2
H&E: hematoxylin and eosin
ICI: immune checkpoint inhibitor
IHC: immunohistochemistry
MDSFR/SFF: multi-diameter single-fibre reflectance/single-fiber fluorescence
nCRT: neoadjuvant chemoradiotherapy
nCT: neoadjuvant chemotherapy
pCR: pathological complete response
PD-L1: programmed death-ligand 1
PD-1: programmed cell death-1
PTS: primary tumor site
qFME: quantitative fluorescence molecular endoscopy
SDS-PAGE: sodium dodecyl-sulfate polyacrylamide gel electrophoresis
THR: tumor-to-healthy tissue ratio
UMCG: University Medical Centre Groningen
USNB/SFF: ultrasound-guided needle biopsy/single-fiber fluorescence
US-qFME: ultrasound-guided quantitative fluorescence molecular endoscopy
