# Supplementary material for: Visualizing the distribution of durvalumab targeting PD-L1 using quantitative fluorescence imaging in patients with esophageal cancer
Source: Theranostics. 2026 Sep 2;16(16):9080–92. doi: 10.7150/thno.136523 (PMC13684177; doi:10.7150/thno.136523)
Supplement: Supplementary file 1 [file thnov16p9080s1.pdf]

SUPPLEMENTARY MATERIAL

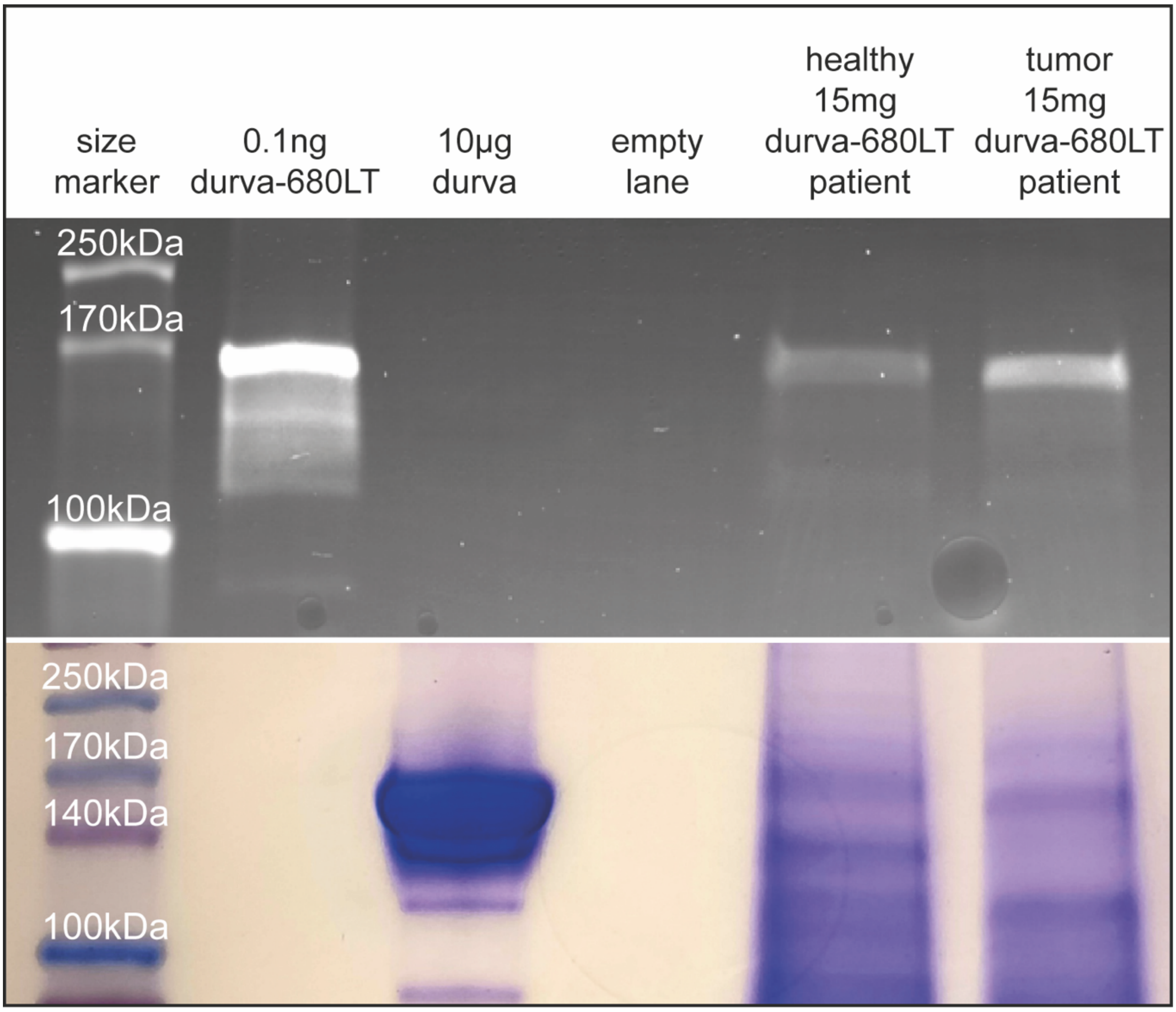

Figure S1: Durvalumab-680LT stability. Fresh-frozen biopsy samples were obtained from one post-CRT patient in the 15-mg durvalumab-680LT group, lysed, and loaded on an SDS-PAGE gel. As positive and negative controls, 0.1 ng durvalumab-680LT and 10 µg unlabeled durvalumab, respectively, were also loaded. The gel was scanned using an Odyssey CLx flatbed scanner (top), then stained overnight with Coomassie blue (bottom).

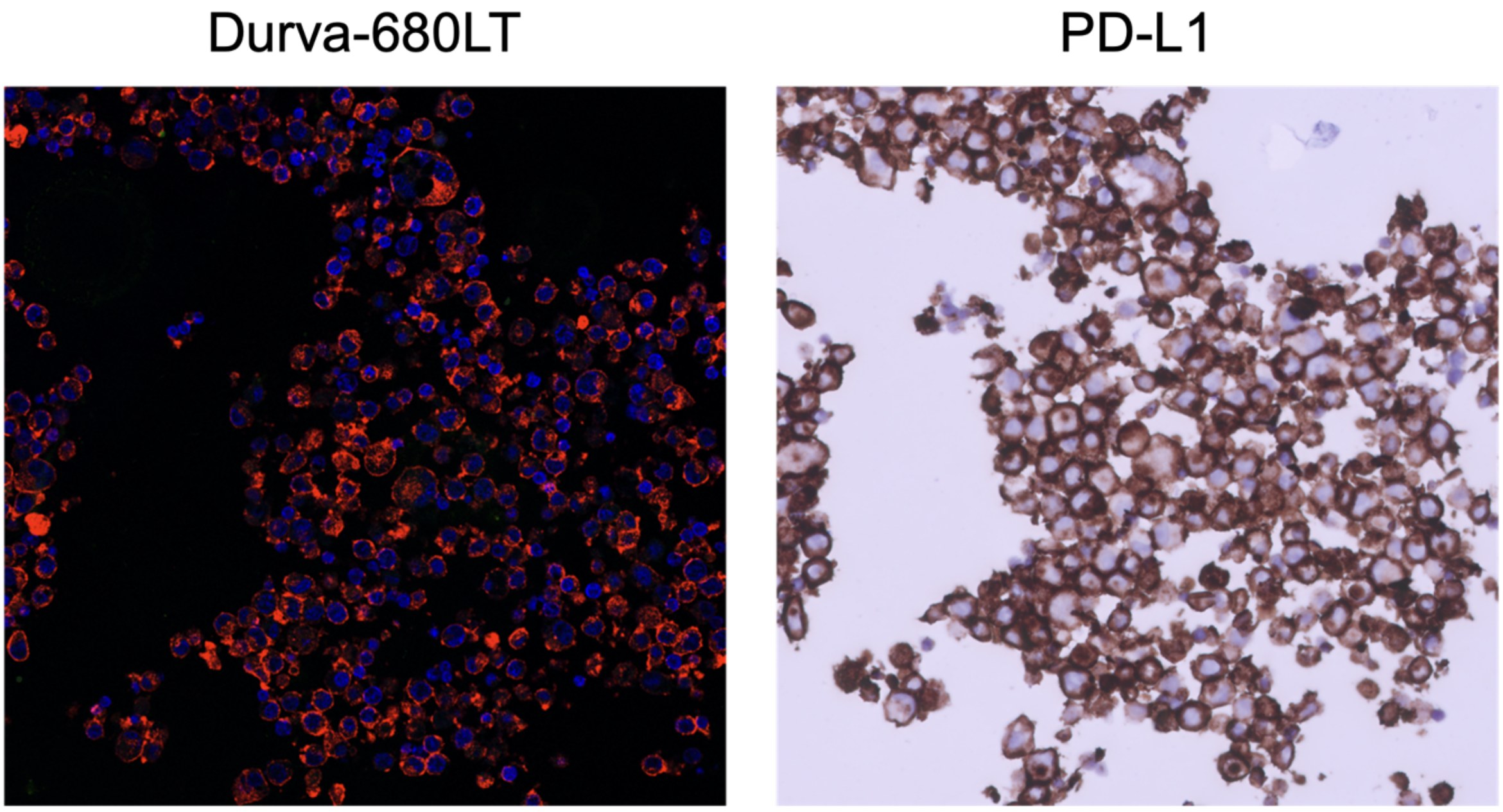

Figure S2: Durvalumab-680LT does not prevent PD-L1 antibody binding measured using immunohistochemistry. RKO cells (a human colorectal cancer cell line) were incubated for 24 hours with 50 nM durvalumab-680LT, fixed, and then embedded in paraffin. (Left) A fluorescence microscopy image showing the durvalumab-680LT signal in red; the nuclei were counterstained with DAPI (blue). (Right) An adjacent section was stained using an antibody against PD-L1. PD-L1: programmed death-ligand 1.
